# Recent Advances in the Management of Diabetic Kidney Disease: Slowing Progression

**DOI:** 10.3390/ijms25063086

**Published:** 2024-03-07

**Authors:** Na Wang, Chun Zhang

**Affiliations:** Department of Nephrology, Union Hospital, Tongji Medical College, Huazhong University of Science and Technology, Wuhan 430022, China; wnflora_2011@163.com

**Keywords:** diabetic kidney disease, chronic kidney disease, molecular mechanisms, biomarkers, therapies

## Abstract

Diabetic kidney disease (DKD) is a major cause of chronic kidney disease (CKD), and it heightens the risk of cardiovascular incidents. The pathogenesis of DKD is thought to involve hemodynamic, inflammatory, and metabolic factors that converge on the fibrotic pathway. Genetic predisposition and unhealthy lifestyle practices both play a significant role in the development and progression of DKD. In spite of the recent emergence of angiotensin receptors blockers (ARBs)/angiotensin converting enzyme inhibitor (ACEI), sodium-glucose cotransporter 2 (SGLT2) inhibitors, and nonsteroidal mineralocorticoid receptors antagonists (NS-MRAs), current therapies still fail to effectively arrest the progression of DKD. Glucagon-like peptide 1 receptor agonists (GLP-1RAs), a promising class of agents, possess the potential to act as renal protectors, effectively slowing the progression of DKD. Other agents, including pentoxifylline (PTF), selonsertib, and baricitinib hold great promise as potential therapies for DKD due to their anti-inflammatory and antifibrotic properties. Multidisciplinary treatment, encompassing lifestyle modifications and drug therapy, can effectively decelerate the progression of DKD. Based on the treatment of heart failure, it is recommended to use multiple drugs in combination rather than a single-use drug for the treatment of DKD. Unearthing the mechanisms underlying DKD is urgent to optimize the management of DKD. Inflammatory and fibrotic factors (including IL-1, MCP-1, MMP-9, CTGF, TNF-a and TGF-β1), along with lncRNAs, not only serve as diagnostic biomarkers, but also hold promise as therapeutic targets. In this review, we delve into the potential mechanisms and the current therapies of DKD. We also explore the additional value of combing these therapies to develop novel treatment strategies. Drawing from the current understanding of DKD pathogenesis, we propose HIF inhibitors, AGE inhibitors, and epigenetic modifications as promising therapeutic targets for the future.

## 1. Introduction

Diabetic kidney disease (DKD), also known as diabetic nephropathy, affecting over 700 million members of the population worldwide, is currently the leading attributable cause of end-stage renal disease (ESRD) [1]. DKD is a serious microvascular sequela that affects approximately 30% of individuals with type 1 diabetes (T1D) and 40% of those with type 2 diabetes (T2D) [2]. In T1D, DKD typically develops after a duration of 10 years with diabetes. However, in T2D, DKD may already be present at the time of diagnosis. In addition to DKD, the microvascular complications of diabetes encompass diabetic neuropathy, diabetic retinopathy, and diabetic foot. The macrovascular complications of diabetes, on the other hand, mainly include cardiovascular diseases (CVD) (including coronary heart disease, heart failure, arrhythmias, sudden cardiac death, cerebrovascular disease, and peripheral artery diseases). T2D, which is the predominant diabetes subtype, accounts for 90–95% of cases. DKD can be categorized into two distinct phenotypes: (1) the classical albuminuric phenotype, which exhibits histological signs of diabetic glomerulopathy, and the non-albuminuric phenotype, characterized by prevalent atherosclerosis, atypical vascular lesions and/or tubulointerstitial fibrosis with a relatively intact glomerular structure. All patients should conduct thorough evaluations of nephropathy (including albumin, estimated glomerular filtrate [eGFR]), neuropathy (incorporating a detailed history, temperature assessment, pinprick sensation, vibration sensation, and monofilament testing), and retinopathy (requiring a comprehensive eye examination) at the initial onset of T2D, and no later than 5 years after the diagnosis of TID, and at least annually thereafter [3,4]. Among them, the diagnosis of diabetic autonomic neuropathy is an exclusive diagnosis, which needs to comprehensively consider the patient’s history of diabetes, clinical symptoms, and related examinations. It is necessary to exclude other causes for neuropathy, including vitamin 12 deficiency, infections, neurotoxic medications, alcohol abuse, hypothyroidism, renal disease, malignancies (e.g., multiplemyeloma), vasculitis, chronic inflammatory demyelinating neuropathy, and inherited neuropathies [5]. Vitamin 12 deficiency, neurotoxic medications, alcohol abuse, and renal disease are the most common additional causes of peripheral neuropathy. The severity of neuropathy are highly associated with the extent of chronic renal insufficiency. While most individuals with chronic renal failure exhibit subtle signs of polyneuropathy, significant neuropathy typically manifests when the creatinine clearance falls below 5 to 6 mL/min or the glomerular filtration rate sinks below 12 mL/min [6]. The neuropathy associated with uremia typically manifests as a distal sensorimotor neuropathy. The exact etiology of the neuropathy of CKD remains unknown, but it is speculated to be a result of the accumulation of a toxin or metabolite. Other potential candidates include parathyroid hormone, methylguanidine, myo-inositol, β2-microglobulin, and calcium [5]. Neurophysiological studies examining nerve excitability before and after dialysis suggest that uremic nerves are in a state of depolarization prior to dialysis, most likely due to hyperkalemia [7]. These studies indicate that potassium may contribute to the development of neuropathy. Furthermore, cardiovascular autonomic neuropathy (CAN) independently predicts the progression of diabetic nephropathy and chronic kidney disease (CKD) in diabetic patients [8]. Other than improved glycemia control, dialysis and rigorous potassium level control may effectively decelerate the progression of diabetic neuropathy. The International Diabetes Federation had estimated that in 2021, 537 million individuals were living with diabetes. The global prevalence of diabetes is projected to reach 600 million by 2035 [9] and further increases to 783 million by 2045, with disproportionate growth in disadvantaged countries [10]. It is estimated that approximately 40% or more individuals with diabetes will progress to develop DKD, with a significant proportion of these individuals ultimately facing kidney failure and requiring dialysis or transplantation. The climbing prevalence of DKD is in parallel with the soaring global diabetes epidemic [2]. Among individuals with T1D or T2D, the presence of DKD significantly elevates the risk of cardiovascular disease. DKD not only confers increased risk of all-cause mortality, but also compromises with low quality of life. Moreover, the number of renal replacement treatment (RRT) recipients is extrapolated to escalate from 2.819 million to 4.35 million by 2035 [11]. The expansion of RRT usage engenders a significant economic burden on the global community. Recognizing DKD at an early stage and promptly addressing risk factors can significantly enhance cost-effectiveness. In spite of the American Diabetes Association’s well-defined screening guidelines, which recommend annual measurements of albuminuria creatinine ration (ACR) and eGFR, DKD remains underdiagnosed in its early stages, posing a significant obstacle to risk-mitigation efforts.

The mechanisms underlying kidney damage in diabetes can be broadly categorized into hemodynamic, inflammatory, and metabolic factors, all of which contribute to the development of fibrosis [12]. The rise in the occurrence of DKD in certain communities may also be linked to epigenetic factors and adverse social determinants of health. Obesity is associated with a higher risk of developing DKD, and it can also accelerate the progression of existing DKD. CKD, whether attributed to diabetes or other causes, is characterized by progressive kidney fibrosis, leading to loss of function. CKD is defined as abnormities of kidney function or structure: e GFR < 60 mL/min/1.73^2^ or the presences of markers of kidney injury, including albuminuria, for more than 3 months. The level of albuminuria is defined as ACR > 30 mg/g or persistent albuminuria (>300 mg/24 h) across multiple measurements over 3 or more months, regardless of eGFR [13].

The pathogenesis of macrovascular complications in patients with T2D is multifaceted and involves various factors. However, insulin resistance and hyperglycemia stand out as the most significant. Insulin resistance is closely associated with obesity in T2D. The key pathological mechanism in the development of macrovascular complications is atherosclerosis, which leads to the narrowing of arterial walls. In obesity patients with T2D, free fatty acids (FFAs) bind to the toll-like receptor (TLR)-4, subsequently leading to a reduction in PI3-kinase (PI3K) and protein kinase B (Akt) activity [14]. This reduction, in turn, diminishes the insulin-mediated glucose transporter type 4 (GLUT-4) expression and activity, as well as impairs responses to insulin binding and activity. Also, the decrease in PI3K and Akt contributes to a reduction in nitric oxide (NO), resulting in the dysfunction of endothelial cells and participating in alterations associated with atherosclerosis [15]. Furthermore, the interaction between FFAs and TLR triggers the activation of the nuclear factor κ light-chain enhancer of activated B cells (NF-κB), which promotes the transcription of inflammatory mediators, leading to insulin resistance and intensifying the atherosclerosis process.

**Figure 1:**
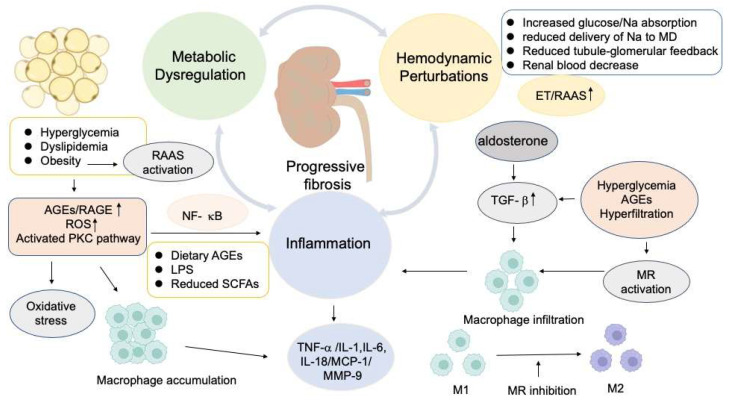
Metabolic, inflammatory, and hemodynamic perturbation pathways in the pathogenesis of DKD. Hyperglycemia, activation of RAAS, and inflammatory responses can lead to hyperfiltration. Increased levels of AGEs/RAGE, ROS, and activated PKC pathway can trigger the accumulation of macrophage and the release of inflammatory factors, eventually leading to kidney fibrosis. The interaction between dietary AGEs and gut microbiota has the potential to trigger an inflammatory response through NF-κB pathway. Hyperglycemia, AGEs, and hyperfiltration can induce macrophage accumulation through upregulating TGF-β and MR. RAAS: renin angiotensin aldosterone system; AGEs: advanced glycation end products; RAGEs: receptors for AGEs; ROS: reactive oxygen species; PKC: polyol and protein kinase C; NF-κB: nuclear factor κ light-chain enhancer of activated B cell; LPS: lipopolysaccharide; SCFAs: short-chain fatty acids; TNF-α: tumor necrosis factor α; IL-1: interleukin-1;IL-6: interleukin-6; IL-18: interleukin-18; MCP-1: monocyte chemoattractant protein-1; MMP-9: matrix metalloproteinase-9; Na: sodium; MD: macula densa; ET: endothelin; TGF-β: transforming growth factor; MR: mineralocorticoid receptor; M1: M1 macrophage; M2: M2 macrophage.

Hyperglycemia boosts the production of reactive oxygen species (ROS), which then inactivate NO, ultimately leading to endothelial dysfunction and atherosclerosis [16]. In parallel to this mechanism, ROS activation results in the activation of protein kinase C (PKC), which plays a crucial role in maintaining vascular homeostasis through various mechanisms, including the regulation of vascular cell growth and apoptosis, as well as the production of different mediators [17]. Endothelin-1 (ET-1), advanced glycation end products (AGEs), receptor for AGEs (RAGE), and cyclooxygenase-2 (COX-2) are also considered to contribute to the progression of atherosclerosis [17,18,19,20]. The macrophages accumulate oxidized lipids, which are from low-density lipoprotein (LDL) particles, ultimately transforming into foam cells. This occurrence subsequently triggers macrophage proliferation and the accumulation of T lymphocytes, which promotes collagen accumulation and muscle proliferation within the vascular wall [21]. The ultimate outcome of this process contributes to atherosclerotic lesions. Albuminuria and reduced eGFR portend increased risk of CVD and all-cause mortality [22]. Cardiovascular mortality is the most prevalent cause of death in patients with advanced CKD (stage 4) and ESRD [23]. Renal fibrosis is an irreversible consequence of DKD, and is triggered by various factors, including renal hemodynamic changes, abnormalities in glucose metabolism, an overactive renin angiotensin aldosterone system (RAAS), ischemia, increased oxidative stress and inflammatory response [24] (Figure 1). Renal fibrosis has a profound impact on tubule, interstitium, glomeruli, vasculature, and other connective tissue, which leads to renal damage, hypoxia, apoptosis, and eventually renal failure. Tubular interstitial hypoxia is thought to be the final common pathway for fibrosis [25]. The foremost strategy for managing DKD is to hinder the progression of fibrosis.

Blood pressure and glycemic control can significantly reduce proteinuria and improve cardiovascular outcomes in patients with DKD. Nothing in addition to blood pressure and glycemic control was available to postpone the progression of DKD until the advent of a trail of renin angiotensin system (RAS) blockade-captopril in populations with type 1 diabetes in 1993 [26]. The RAS blockers were further consolidated in patients with diabetes during the subsequent 8 years [27,28]. Although angiotensin receptor blockers (ARBs) were estimated to halt the progression of DKD by 5–7 mL/min/year [26], recent research revealed that the progression of DKD still persisted. In 2014, SGLT2 inhibitors were discovered unexpectedly to further prevent the progression of DKD and multiple outcome trials solidified the salutary effects of SGLT2 inhibitors in DKD populations [29]. The primary mechanism by which SGLT2 inhibitors offer nephroprotection is by enhancing distal sodium delivery and stimulating tubule-glomerular feedback. This leads to afferent vasoconstriction, ultimately reducing intraglomerular pressure. Almost around the same time of the discovery of SGLT2 inhibitors, innovative research on NS-MRAs, specifically finerenone, also were initiated [30]. This drug not only slowed the progression of DKD, but also achieved remarkable efficacy. GLP-1RAs were recommended for DKD patients to gain better glycemic treatment even after optimizing treatment with SGLT2 inhibitors [31]. CV outcome trails (CVOT) had convincingly demonstrated that GLP-1RAs significantly reduced cardiovascular events [32]. A post hoc analysis of renal outcomes from CVOT revealed a notable protective effect of GLP-1RAs [33]. The randomized placebo-controlled trail (FLOW) was initially designed to assess the efficiency of semaglutide in individuals with T2D and CKD, with a primary emphasis on renal outcomes [34]. It was subsequently announced that the trial would be stopped early due to the results from an interim analysis meeting the certain preassigned criteria. Other agents including pentoxifylline (PTF), selonsertib, and baricitinib, hold significant promise as potential therapies for DKD. The current medications used to treat DKD can effectively mitigate renal fibrosis through multiple mechanisms, including controlling blood glucose levels, inhibiting the inflammatory response, antioxidant stress, and regulating cytokines (specifically, the transforming growth factor) and vascular endothelial growth factors (VEGF). The pursuit of creating precise medicine that effectively targets inflammation and the fibrosis pathway is currently under investigation.

Once DKD and its adverse pathophysiological changes have begun, regression occurs extremely infrequently. Albuminuria and eGFR are the most prominent clinical indicators in DKD. Novel biomarkers for the early diagnosis or prognosis of DKD are of great importance. In essence, the management of DKD is to decelerate the progression of kidney disease and forestall cardiovascular complications. Albuminuria reduction, precise glycemic control and blood pressure regulation are surrogate endpoints that can be targeted to delay the advancement of DKD. Despite the current therapies available, the progression of DKD remains unabated. This suggests that more effective treatment opinions are necessary to effectively manage and potentially reverse the course of this condition. Research is actively ongoing to gain a deeper understanding of the underlying mechanisms of DKD and to develop innovative therapeutic strategies that can effectively halt or even reverse its progression. In this review, we present an overview of the current understanding of the pathophysiological mechanisms of DKD, and we discuss the latest evidence-based interventions, including the promising agents -GLP-1RAs. In addition, we have also compiled a comprehensive list of potential biomarkers and therapeutic agents for DKD, which may serve as valuable resources for future research. By doing so, we hope to pave the way for the development of innovative treatment options.

## 2. Molecular Mechanisms of Kidney Damage in Diabetes

### 2.1. Glomerular Hemodynamic Perturbations in the Pathogenesis of DKD

Hyperglycemia, if left untreated, can trigger glomerular hyperfiltration and hypertension. The hemodynamic effects are vital to maintain the glomerular homeostasis and surround the RAAS (Figure 1). SGLT2 is recognized as a crucial regulator of glomerular hemodynamics. The expression of SGLT2 on the luminal surface of epithelial cells in the proximal convoluted tubule serves as a crucial mechanism for enhancing the reabsorption of proximal tubular Na and glucose. This process serves to suppress the tubule-glomerular feedback by reducing the delivery of sodium chloride (NaCl) to the macula densa [35]. However, it may also contribute to the deterioration of hyperglycemia [36]. The reduction in tubule-glomerular feedback can result in the dilation of the afferent arteriole and the elevation of angiotensin II in the efferent arteriole, ultimately leading to vasoconstriction [35]. Furthermore, SGLT2 inhibitors assist in restoring tubule-glomerular feedback by enhancing the distal delivery of sodium chloride to the macula densa, where solute reabsorption produces adenosine, a by-product of ATP utilization. Adenosine exerts its actions in a paracrine manner, potentiating afferent arteriolar vasoconstriction and suppressing renin release from juxtaglomerular cells [12]. Additionally, it may also contribute to the reduction in efferent arteriolar constriction. The vasodilatation of afferent arteriole and the vasoconstriction of efferent arteriole can lead to hyperfiltration, which is widely recognized as the initial step in the development of DKD [2]. Hemodynamic disturbances play a pivotal role in the aberrant activation of endothelin (ET). Endothelin has the ability to regulate renal flow blood and glomerular filtration rate [37], potentially leading to vasoconstriction effect within the renal vasculature. Dyslipidemia, hyperglycemia, endothelial dysfunction, and oxidative stress all contribute to the elevation of plasma ET [38]. Endothelin receptor (ER) blockade has been demonstrated to effectively reverse the progression of CKD [39]. This finding highlights the critical role of endothelin in the development and progression of CKD, and offers hope for new treatment options that can halt or even reverse the course of this debilitating condition. The cyclo-oxygenase 2 (COX-2) derived prostanoids, expressed in endothelial cells within the renal tissue, have been considered to regulate renal auto-regulatory functions at the macula densa and mediate the dilation function of afferent arteriole [40], ultimately resulting in hyperfiltration. The glomerular hyperfiltration process results in progressive albuminuria, a gradual reduction in the eGFR, and finally the development of ESRD [41]. Hyperglycemia, glomerular hypertension, and elevated amino acid levels can exacerbate glomerular hyperfiltration [35], leading to potential renal damage. Hemodynamic mechanisms have long been recognized to initiate and propagate kidney injury through resulting in glomerular hypertrophy, which can ultimately contribute to glomerulosclerosis and a loss of renal function.

#### The Activation of Renin-Angiotensin-Aldosterone System (RAAS) in DKD

The RAAS participates in the development and progression of DKD [42]. The renin is produced by the juxtaglomerular cells of the nephron and is found to be contiguous to the afferent arterioles. The renin plays a pivotal role in initiating the RAS, which generates more intense vasoconstriction in the efferent arteriole compared to the afferent arteriole [43]. Angiotensin converting enzyme 2 (ACE2) plays important roles in the dilation of glomerular afferent arterioles through converting angiotensin II into angiotensin 1–7. Produced using the activation of RAS, angiotensin II binds to specific receptors, namely AT1 and AT2. The activation of AT1 modulates the elevated resistance of the efferent arteriole [44], contributing to hyperfiltration, and activation of AT2 exerts a protective counterregulatory role in renal flow, including prostaglandin release and regulating renal vasodilation [45]. The interaction between angiotensin II and its receptors leads to a cascade of physiological responses that regulate blood pressure and fluid balance. High levels of angiotensin II accelerate renal damage through several mechanisms, including modulating calcium influx into podocyte [46], stimulating the expression of proinflammatory cytokines (tumor necrosis factor α [TNF-α], interleukin [IL-1, IL-6, IL-18], monocyte chemoattractant protein-1 [MCP-1]), matrix metalloproteinase-9 [MMP-9] and profibrotic mediators (transforming growth factor [TGF-β]) [12,47], macrophage activation [48], and increased secretion of adrenal aldosterone. Adrenal aldosterone has the potential to upregulate the expression of profibrotic factors, including TGF-β, which boost macrophage infiltration and promote the development of fibrosis of the kidneys [49].

### 2.2. Inflammatory and Fibrotic Factors Involving in DKD

Inflammation and fibrosis are two dominant and interrelated factors that promote the progression of DKD. Hyperglycemia triggers a cascade of intracellular processes that contribute to kidney damage via inflammation and fibrosis (Figure 1). Growth factors, inflammatory cytokines, and chemokines have been substantiated to be elevated in renal biopsy samples from patients with DKD [50]. Substantial components of the immune system, including circulating leukocytes, chemokines, and cytokines, are activated in diabetes [51]. The altered intracellular glucose metabolism leads to the generation of AGEs and ROS, and the activation of PKC and the Janus kinase (JAK)-signal transducer and activator of the transcription (STAT) signaling pathway [52]. The pathological variations of DKD are characterized by nodular and diffuse mesangial expansion, thickening of the glomerular and tubular basement membranes, as well as podocyte damage and detachment, which are attributed to sustained glomerular hypertension and hyperfiltration, subsequent to tubular atrophy and glomerular sclerosis, and eventually an apparent decline in renal function [53].

TNF-α is produced by activated macrophages and resident kidney cells in glomerular and tubular membranes, which plays a vital role in evoking chemokines, cytokines, cytotoxic effects, and apoptosis [12]. The activation of NF-κB can lead to the production of inflammatory factors, including TNF-α, which can accelerate the progression of DKD [54]. Diabetic cohorts revealed that the TNF-α receptor superfamily members were related to a high risk of ESRD in diabetes [55]. The cytokines, including IL-1, IL-6, IL-16, and IL-18, have been shown to be involved in the pathogenesis of DKD. IL-1 can cause hyperpermeability of endothelial cells and excessive glomerular blood flow through promoting the release of phospholipase A2 and prostaglandin E [56]. Infiltrating macrophages and hyperglycemia both contribute to the release of IL-1β, a superfamily of IL-1, which is intimately involved in the pathogenesis of DKD [57]. IL-6 recruits neutrophil infiltration in the tubulointerstitium, which is correlated to podocyte hypertrophy, and GBM thickening [12], eventually resulting in albuminuria and a decrease in renal function. The injection of IL-6 neutralizing antibody into diabetic mice resulted in a prominent reduction in collagen and fibrosis by ameliorating mesenchymal transition [58]. IL-18 instigates the unleashing of interferon-γ, the expression of adhesion molecules and apoptosis [56]. The expression of IL-18 in renal tissues is intimately associated with the development of albuminuria in patients with DKD [56]. Microalbuminuria, if left untreated, can progress to macroalbuminuria or overt proteinuria, resulting in a significant decline in eGFR. A decline in eGFR can ultimately lead to uremia. In patients with vascular disease, albuminuria changes independently predict mortality, cardiovascular events, and renal outcomes.

MCP-1, also referred to as CC chemokine ligand 2, has been confirmed to elevate in kidney biopsies from patients with DKD, which may elicit inflammatory cell recruitment, migration, and the interaction of inflammatory cells, finally contributing to kidney injury [59,60]. MMP-9, expressed in the proximal renal tubular epithelial cells, has been validated to regulate the degradation of the extracellular matrix during renal fibrosis [61]. The downregulation of MMP-9 can effectively slow the progression of DKD by improving creatinine and reducing proteinuria [62].

Kidney damage in diabetes is pronouncedly featured by monocytes and macrophages. The amassment of macrophages exhibits a close relationship with the histological severity of kidney disease in diabetes [59,63]. Macrophages can exacerbate kidney injury by modulating tissue repair and fibrosis [64,65]. Hyperglycemia, angiotensin II, endothelial cell dysfunction, oxidized low-density lipoprotein (LDL), and AGEs promote the accumulation of macrophages [48]. M1 macrophages have the potential to transition into an anti-inflammatory M2 macrophage [64]. Upon kidney injury, M1 macrophages secrete substantial inflammatory factors, including IL-1, IL-6, MMP-9, and TNF-α [64]. Macrophage infiltration during acute kidney injury may facilitate a transition to chronic injury. The balance between M1 and M2 macrophages remains a significant challenge in the development of macrophage-based therapy for DKD.

Tubulointerstitial fibrosis marks the irreversible outcome of advancing kidney disease, intricately correlated to extracellular matrix accumulation and tubular atrophy [66]. TGF-β, expressed ubiquitously by kidney cells, infiltrates macrophages and leukocytes, creates pleiotropic effects that span immunomodulation, angiogenesis, and extracellular matrix formation in the progression of kidney diseases. TGF-β acts as a master mediator of DKD via regulating inflammation and fibrosis [67]. This regulatory function of TGF-β is further supported by its downstream signaling molecules, the Smads in the progression of DKD. Specifically, Smad3 has been shown to foster autophagy dysregulation by provoking lysosome depletion in the tubular epithelial cells of DKD [68]. Additionally, a recent study has identified leucine-rich -2-glycoprotein 1 (LRG1) as a factor that exacerbates kidney fibrosis by augmenting TGF-β/Smad3 signal transduction [69]. Klotho, which is primarily expressed in kidney cells, has been reported to be a potential therapeutic approach for DKD through regulating calcium and phosphate metabolism, reducing apoptosis, guarding against oxidative stress, and playing anti-inflammatory and antifibrotic roles [70].

Angiotensin II-mediated reactive oxygen species (ROS) or protein kinase C (PKC) and p38 mitogen-activated protein kinase can trigger CTGF. Additionally, the plasminogen activator inhibitor (PAI-1) can be activated by TGF-β [71,72]. PAI-1accelerates kidney fibrosis by restraining the production of plasmin from plasminogen, which maintains the extracellular matrix accumulation.

Hyperglycemia, AGEs and glomerular hypertension can upregulate the expression of TGF-β [73]. Fibronectin is shown to result in mesangial expansion and the deterioration of albuminuria, ultimately contributing to exacerbated kidney function [74]. The treatment of DKD with mesenchymal stem cell therapy can effectively diminish fibronectin levels, improving renal function and albuminuria [75]. Furthermore, metformin has been reported to reduce collagen-1 levels together with fibronectin [76]. Studies reveal that collagen-1 propels the progression of renal fibrosis and the overabundant accumulation of the extracellular matrix in DKD [77]. The precise mechanism of collagen-1 in the pathogenesis of DKD remains elusive and requires further exploration. The serine/threonine kinase, known as an apoptosis signal-regulating kinase 1 (ASK1) induced by oxidative stress, evokes apoptosis, inflammation, and fibrosis [78]. ASK1 has been incriminated to participate in the pathogenesis of DKD through phosphorylating and activating c-Jun N-terminal kinase (JNK) and p38 mitogen-activated protein kinase [79]. Additionally, glucose dysmetabolism can activate PKC and the JAK-STAT pathways [52]. The JAK-STAT pathway prompts the expression of proinflammatory factors and multiple chemokines, enhancing the inflammatory response in DKD [59]. The JAK-STAT is highly expressed in the glomeruli and tubulointerstitial cells in a population with T2D, and there is an inverse relationship between its activity and eGFR [80]. These mechanisms suggest potential therapeutic targets for the treatment of DKD.

CTGF is found to be associated with tubulointerstitial fibrosis and glomerulosclerosis in various renal diseases [71]. Urinary CTGF concentration is related to a high risk of albuminuria and decreased eGFR [71]. The synthesis of fibronectin and type 1 collagen is elevated when mesangial cells are exposed to CTGF [71]. Additionally, a phosphatase and tensin homolog (PTEN) has been identified as a factor that increases the risk of decreased eGFR of DKD patients [81]. Furthermore, PTEN has been found to potentiate the expression of IL-6 and CTGF [82]. These findings provide valuable insights into the mechanisms underlying the development and progression of renal disease, particularly in individuals with diabetes.

### 2.3. The Significance of Metabolic Factors in the Progression of DKD

Hyperglycemia, increased adiposity and dyslipidemia can all enhance the overproduction of vasoactive mediators, including AGEs and ROS [83,84] (Figure 1). Upon interacting with RAGEs, AGEs lead to fibrosis and irreversible progression in DKD through distorting the extracellular matrix architecture and regulating cellular functions [85]. It is important to note that RAGEs are detected throughout the kidney. The accumulation of AGEs in the renal basement can upregulate the expression of RAGE on podocytes in DKD, ultimately inducing NF-κB mediated inflammation, fibrosis, and oxidative stress [86]. In podocytes and endothelial cells, AGEs attach to RAGE, which triggers inflammation via the nucleotide-binding oligomerization domain-like receptor pyrin domain containing 3 inflammasome [87]. Furthermore, AGEs enhance the expression of serum amyloid A, another RAGE activator, leading to a self-perpetuating feedforward cycle of inflammatory gene expression [59]. These intracellular signals result in the continuous release of proinflammatory mediators, profibrotic factors, and immune cell recruitment. The RAGE inhibitor has been reported to effectively hinder the progression of DKD in rats [88]. The potential therapeutic benefits of targeting AGEs or the AGE/RAGE axis in DKD are promising. AGEs also can contribute to impaired vasodilatation in diabetes through suppressing the bioavailability of endothelium-derived NO and elevating the production of ROS [89]. ROS accelerate the progression of DKD through podocytes apoptosis and the process of epithelial to mesenchymal transition (EMT) [84,90]. ROS are generated by the nucleotide-binding domain leukin-rich repeat-containing family pyrin domain-containing 3 (NLRP3) inflammasomes, and which are activated by hyperglycemia [90]. Recent research has shown that knocking down NLRP3 can impede podocytes injury through reducing the hyperglycemia-induced production of mitochondrial ROS in renal mesangial cells and preventing lipid accumulation [91]. This suggests that targeting NLRP3 may offer a potential therapeutic approach for DKD. The overexpression of the pro-oxidant enzyme NADPH oxidase 5 (NOX 5) is demonstrated to promote albuminuria, inflammation, and renal fibrosis in diabetes by increasing ROS formation [91]. Additionally, hyperglycemia can also modify the polyol and PKC pathways, leading to diminished endothelial nitric oxide synthase and amplifying oxidative stress, respectively, resulting in higher vascular endothelial growth factor and endothelin levels [48]. Hyperglycemia, dyslipidemia, and insulin resistance are the common features of diabetes and can potentiate vicious cycle of inflammatory and oxidative processes [59,92].

### 2.4. The Impact of Dietary AGEs and Gut Microbiome Variation on the Progression of DKD

AGEs exposure can partly result from diet as well as hyperglycemia [93]. AGEs contribute to glomerular pathological alterations, including glomerular hypertrophy, glomerular basement membrane widening, mesangial expansion, and glomerular sclerosis [94]. Furthermore, dietary AGEs interact with gut microbiota, evoking local inflammation and the release of inflammatory factors [95]. AGEs-rich foods can disrupt the intestinal mucosal barrier and allow the translocation of inflammatory mediators into systemic circulation, causing local kidney inflammation [96]. The gut microbiota can perceive molecules produced by the host. For instance, the release of norepinephrine in response to stress can enhance the growth and production of virulence-associated factors in Gram-negative bacteria [97]. As the progression of DKD, uremic toxins result in relocation towards Gram-negative bacteria in the gut, such as *Klebsiella oxytoca*, *Bifidobacterium*, *Turicibacter*, and *Allobaculum* genera [98,99]. Endotoxin, which acts as the hydrophobic anchor of lipopolysaccharides, is a phospholipid that forms in the outer membranes of most Gram-negative bacteria. It is constantly produced in the gut and is then transported into the intestinal capillaries through the TLR-4-depent mechanism [100]. Gut microbiota-derived phenyl sulfate has been reported to lead to podocyte injury and albuminuria. Endotoxemia is a potential cause of inflammation in patients with CKD. The LPS from the cell wall of Gram-negative bacteria binds to TLR-4, leading to an increase in local cytokine production, recruitment of inflammatory cells and the release of LPS [101]. Upon exposure to TLR-4 on podocytes or other kidney cells, LPS potentiate inflammation and fibrosis, ultimately resulting in podocyte damage, tubular injury, glomerular hypertrophy, and hypercellularity, as well as albuminuria in STZ-induced diabetic mice [102]. Alteration in the gut microbiota has been incriminated in the pathogenesis of DKD. Additionally, a reduction in dietary-associated short-chain fatty acids (SCFAs) from the gut microbiota also exacerbates podocyte damage, interstitial fibrosis, and albuminuria by promoting epithelial cell dysfunction and gut inflammation [103].

### 2.5. Genetic Predisposition and Epigenetic Modifications in the Progression of DKD

The inherent progression of DKD, coupled with indirect diagnostic methods (such as albuminuria and eGFR), as well as the presence of diverse risk factors, has greatly complicated the definition of DKD as a genetic phenotype. This complexity has probably led to inconsistent and limited findings in genetic analysis. Among the probands with diabetes, those who had siblings with DKD faced a significantly higher risk of developing DKD themselves, ranging from approximately 2- to 4-fold higher than those who had siblings with diabetes but without DKD [104]. There was a strong familial clustering of DKD in both T1D and T2D. The heritability analysis of DKD has estimated that 34–59% of the variance in DKD in individuals with T1D (after adjusting for factors such as sex, age at diabetes diagnosis, and diabetes duration) can be attributed to common genetic variants (24–42% unadjusted), depending on precise definition of DKD including both albuminuria and eGFR levels [105]. It is worth noting that a similar unadjusted analysis of DKD in individuals with T2D has estimated SNP heritability to be only 8%, presumably due to the significantly greater phenotypic heterogeneity of kidney disease in T2D compared to T1D [106]. Fine mapping in multiethnic populations has identified an indel in exon 2 of the *CNDP1* gene, which is associated with both DKD and serum carnosinase levels [107]. This finding suggests that the *CNDP1* gene may play a role in the development of DKD and regulation of serum carnosinase levels. Carnosine is a dipeptide that has antioxidant and anti-inflammatory properties through blocking the glucose-induced increase in collagen on podocytes, extracellular matrix components’ fibronectin, and TGF-β in mesangial cells [108]. Serum carnosinase levels can be used as a biomarker for various diseases. Therefore, understanding the genetic basis of serum carnosinase levels may provide insights into the pathogenesis of DKD and other diseases. One promising discovery, supported by robust statistical and functional evidence, links EPO (erythropoietin) promoter polymorphism to both the presence of proliferative diabetic retinopathy (PDR) and ESRD, as well as EPO expression [109].

Although kidney disease occurs at equal rates in individuals with T1D and T2D (30~40%), the presence of high rates of co-occurring kidney disease risk factors (such as high blood pressure and obesity) among individuals with T2D results in increased phenotypic heterogeneity in patients with T2D and DKD, making genetic discovery more challenging. The majority of genome-wide significant loci identified through a genome-wide association study (GWAS) of DKD have been discovered utilizing T1D cohorts. The first GWAS study of DKD in individuals with T1D with or without kidney disease (defined as the presence of macroalbuminuria or ESRD for at least 15 years without clinical evidence of kidney disease) have successfully identified two genome-wide significant loci that are associated with ESRD, specifically in patients who have ESRD compared to those with T1D but without ESRD [110]. The first locus, SNP rs12437854, is located on chromosome 15q26 within a large gene desert between the *RGMA* and *MCTP2* genes. The second locus, intronic SNP rs7583877, is found on chromosome 2q11 within the *AFF3* gene, which has been shown to be upregulated in renal endothelial cells when stimulated by pro-fibrotic TGF-β1. Two neighboring SNPs in *ERBB4* are found to be associated with the allele-specific expression of *ERBB4* in the tubulointerstitial tissue of T2D among Pima Indians with DKD [110]. The SNP rs7588550, located within intron 1 of the *ERBB4* gene, is found to have a significant association with DKD (P = 2.1 × 10^−7^). This finding supports the potential role of *ERBB4* in the development of DKD. However, further studies are needed to confirm these results and to explore the underlying mechanisms of this association.

In 2019, a large GWAS on DKD was conducted on individuals of European ancestry with T1D, and 16 novel genome-wide significant loci associated with various disease definitions were identified by the GENIE consortium [111]. The most significant association was observed for SNP rs55703767, a common missense variant located within exon 17 of the type IV collagen alpha 3 chain (*COL4A3*). Notably, the minor allele (T) of this SNP serves as a protective factor against the development of DKD in individuals with T1D and various other albuminuria-related phenotypes. This particular variant of *COL4A3* is linked to reduced GBM thickening and glomerulosclerosis, particularly among patients with either T1D or T2D who have undergone kidney biopsy and have genetic data available. The protective association was particularly evident in individuals with elevated haemoglobinA1c (HbA1c) levels (>7%). Notably, missense mutations in the *COL4A3* gene, which encodes a significant structural component of the glomerular basement membrane (GBM), have been well-documented to cause Alport syndrome [111]. This finding is of particular importance in understanding the pathogenesis of this condition and potentially developing novel treatment options. Furthermore, another three genes (*COLEC11*, *DDR1*, *COL20A1*) are related to collagen pathophysiology and kidney fibrosis. The SNP rs144434404, located in intron 1 of *BMP7*, a gene involved in renal development, is almost exclusively expressed in podocytes in mice [111]. This variant is found to be associated with microalbuminuria, which is characterized by the presence of small amounts of protein in the urine. The SNPs rs142823282, located near *TAMM41*, and rs145681168, found within intron 3 of *HAND2-AS1*, are both strongly associated with microalbuminuria at a study-wide level of significance [111]. The *TAMM41* signal is also linked to the expression of the nearby gene *PPARG*, which is a well-known T2D GWAS gene. However, the association of *PPARG* with DKD has yet to be firmly established.

In 2018, genetic studies of DKD were conducted to identify one genome-wide significant locus (SNP rs9942471) associated with microalbuminuria in ~27,000 individuals of European ancestry with T2D (~13,000 with DKD) [106]. The major allele of SNP rs9942471 is associated with reduced *GABRR1* expression. The *GABRR1* gene variant is strongly associated with the presence of microalbuminuria [106]. This gene is upregulated in glomerular diseases characterized by inflammation and fibrosis. Furthermore, two loci (*UMOD* and *PRKAG2*), which have been previously associated with eGFR in the general population, are also found to be associated with eGFR in individuals with combined T1D and T2D in ~31,000 patients of European and Asian descent [106]. Both *SCAF8* and *CNKSR3* are associated with DKD in individuals of European American, American Indian, and Mexican Indian ancestry with T1D and T2D [112]. Three genes (*ENPP7*, *GNG7*, and *APOL1*) are identified to be significantly associated with ESRD without diabetes in African American individuals [113]. In contrast to protective genetic variants, the *APOL-1 G1/G2* alleles, particularly in individuals of African descent, have the potential to facilitate the development and progression of nondiabetic CKD, which is attributed to a “second hit”. Recently, a new variant of *APOL-1* (rs9622363) has been identified in a large-scale genome-wide association study meta-analysis, specifically among African American individuals with T2D. This discovery has been linked to an increased risk of kidney failure, suggesting that this variant may play a role in the development and progression of DKD. Epigenetic modifications, including DNA methylation, play a crucial role in modulating the impact of the genotype on the development of DKD. Comprehensive analysis integrating genetics and epigenetics from a cohort of individuals with DKD identified distinct cytosine methylation alterations that regulate immune function and inflammation, including the clearance of apoptotic cells by macrophages and complement activation [114]. Genetic studies have encountered challenges due to the limited availability of large datasets for individuals with genotyping and the diverse presentations of DKD. Linking genetic characteristics with DKD is a crucial step towards a deeper comprehension of disease susceptibility and the identification of potential treatment targets. Controlling hyperglycemia only moderately lowers the risk of DKD onset or progression in individuals with long-term diabetes. Previous hyperglycemia can result in long-lasting epigenetic modifications, including acetylation and histone methylation, which subsequently lead to the upregulation of proinflammatory and profibrotic genes [115]. This effect is called “metabolic memory”. As a result, the initial activation of pathways by metabolic disturbances may lead to a self-perpetuating status.

Recently, long noncoding RNAs (lncRNAs), which constitute the principal class of noncoding RNAs, are also considered to paly crucial roles in the pathophysiology of DKD. The altered expression levels of lncRNAs significantly contribute to the development of proteinuria and the associated DKD [116]. LncRNAs play a pivotal role in the development and progression of kidney disease by regulating numerous critical factors, such as pathological processes in mesangial cells, podocytes, and reactive oxidative species, as well as the transition from epithelial to mesenchymal (EMT) and endothelial to mesenchymal (EndMT) [117,118]. Additionally, lncRNAs can modulate microRNAs, further adding to their regulatory complexity. LncRNAs (including plasmacytoma variant translocation 1 [*PVT1*], and metastasis-associated lung adenocarcinoma transcript 1 [*MALAT1*]) are associated with the progression of kidney disease [119,120]. In brief, lncRNAs contribute to the pathogenesis of DKD by potentiating oxidative stress, inflammation, and fibrosis [117]. LncRNAs exhibit remarkable stability in biological fluids, making them promising candidates for potential biomarkers in various diseases. Unlike other RNAs, lncRNAs are stable in these environments, offering a unique opportunity for their utilization in clinical applications.

## 3. Targeting Mechanisms and Recent Advances in the Therapy of DKD

**Figure 2:**
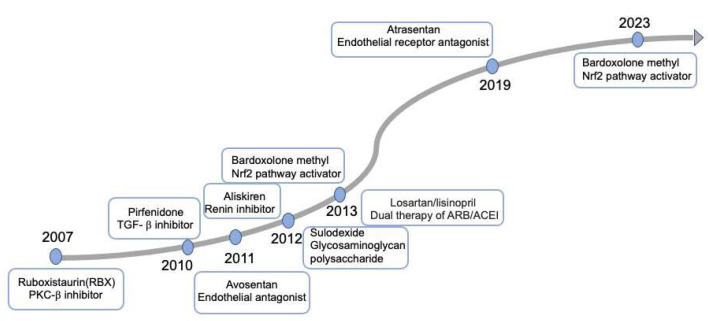
The timeline of major therapy targeting precise molecular processes of DKD. Since 2007, numerous clinical trials of therapies targeting precise molecular processes have been attempted, yet they consistently failed to achieve success due to inefficiency or severe side effects. As a result, these agents failed to be adopted for the treatment of DKD. PKC: polyol and protein kinase C; TGF-β: transforming growth factor; MR: mineralocorticoid receptor; Nrf2: nuclear 1 factor (erythroid-derived 2)-related factor 2; ACEI: angiotensin converting enzyme inhibitor; ARB: angiotensin receptor blockers.

The underlying molecular mechanisms play vital roles in the development of effective therapies to reduce the onset and progression of DKD. Despite attempts to use precision medicine by connecting these molecular mechanisms with therapeutic strategies, they have ultimately failed to be adopted for DKD, as illustrated in Figure 2. The long-term diabetic retinopathy study of ruboxistaurin(RBX), a PKC-beta (PKC-β) inhibitor, failed to prevent kidney outcomes [121]. The ASCEND study of the endothelial antagonist (EA) avosentan was reported to reduce albuminuria but increase fluid retention and heart failure [122]. In 2019, the SONAR trial evaluated the reno-protective effect of atrasentan, a selective endothelial receptor antagonist (ERA), which drew a similar conclusion (hospitalization, anemia, and fluid retention) with avosentan [123]. Given the uncertain benefits and potential risks of heart failure associated with ERAs, they are not recommended for DKD patients according to the KDIGO 2022 Guideline for Diabetes Management [124]. In 2011, a small scale of RCT of pirfenidone, an oral antifibrotic and anti-inflammatory agent, was conducted to assess primary renal outcomes. However, the conclusion from this study was incompletely ascertainable [125]. Currently, a phase II study of pirfenidone for renal fibrosis is ongoing (NCT04258397) and will be completed by 2024. Additionally, sulodexide, a mixture of glycosaminoglycan polysaccharide components, was demonstrated to exhibit no renoprotective effect on patients with type 2 diabetes, macroalbuminuria, and renal impairment [126]. The trial of aliskiren, a renin inhibitor, was discontinued prematurely and demonstrated to be even harmful [127]. Bardoxolone methyl exerted antioxidant capacity and anti-inflammation through activating the Keap1-nuclear 1 factor (erythroid-derived 2)-related factor 2 (Nrf2) pathway [128]. However, the trail of Bardoxolone methy, did not exhibit a reduced risk of ESRD, and was terminated due to a greater rate of cardiovascular events [128]. Following this study, a phase 2 TSUBAKI study revealed an improved eGFR and no incidence of cardiovascular events [129]. Subsequently, a new large multicenter phase 3 study (AYAME) was conducted in Japan to investigate the long-term efficacy and safety of bardoxolone methy [130]. Unfortunately, the clinical trial was terminated, which indicted that bardoxolone methy failed to be used for DKD.

**Figure 3:**
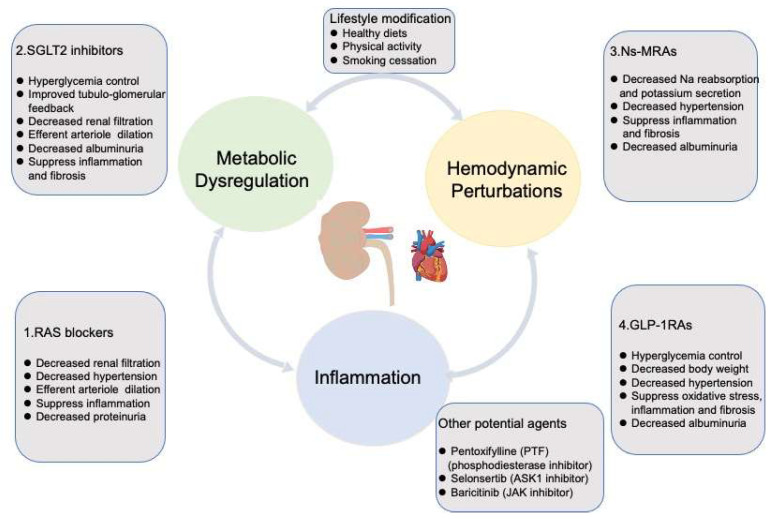
Recent advances in the therapy and potential mechanisms for slowing the progression of DKD. Multidisciplinary treatment including lifestyle modification with RAS blockers, SGLT2 inhibitors, Ns-MRAs, and GLP-1RA are recommended to be adopted for DKD. Meanwhile, the clinical trials of PTF, selonsertib, and baricitinib have exhibited potential for the treatment of DKD. However, more RCTs are required to further evaluate their effectiveness. RAS: renin-angiotensin/aldosterone system; SGLT2: sodium-glucose cotransporter 2; Ns-MRAs: nonsteroidal mineralocorticoid receptors antagonists; GLP-1RAs: glucagon-like peptide 1 receptor agonists; ASK1: apoptosis signal -regulating kinase 1; JAK: Janus kinase.

Since 2001, ARBs have demonstrated a clear benefit in preventing the progression of DKD. However, the subsequent novel therapies mentioned previously failed to show advantages until the appearance of SGLT2 inhibitors in 2014. With the approval of the NS-MRA-finerenone in 2021, we have gained more confidence in managing DKD effectively. According to an interim analysis of RCT (FLOW), GLP-1RA is beginning a new era for DKD. In this section, we mainly focus on the drugs whose clinical effects have been proved as therapeutic agents for DKD (Figure 3). We will also consider the pillars of therapy, primarily adopted by cardiologists in the treatment of heart failure, to determine when and how to administer these drugs in a way that will maximally reduce the progression of DKD.

### 3.1. Renin-Angiotensin/Aldosterone System Blockades

RAASi (ACEI or ARBs) are the preferred antihypertensive agents for patients with DKD, particularly those with evidence of albuminuria (Figure 3). The first RCT of captopril, an ACEI, was performed to evaluate the renoprotective properties in slowing down the progression of DKD. Treatment with captopril displayed notable reduction in the risk of experiencing a doubling of serum creatinine (Scr) as a primary outcome and composite end points (death, transplantation, and dialysis) as a secondary outcome, which was independent of blood pressure management in type 1 diabetic nephropathy [26]. However, type 2 diabetic nephropathy achieved no additional benefits from captopril. It has been discovered that zofenopril, another ACEI with the sulphydryl group (captopril), effectively reduced arterial stiffness compared to enalapril [131]. Zofenopril was confirmed to increase the availability of NO and reduce endothelin-1 production [132]. A post hoc analysis of doubled-blind randomized studies has shown that using zofenopril alone or in combination with a thiazide diuretic, effectively manages BP, particular in patients with hypertension and compromised kidney function [133,134]. The long-term administration of the zofenopril-plus-hydrochlorothiazide combination in high-risk hypertensive patients led to a slight enhancement in urine protein excretion, which was similar to that observed for the combination of an ARB (irbesartan) with a thiazide diuretic. In addition, the percentages of patients with reduced creatinine clearance (<60 mL/min) was minimal and remained consistent between the baseline and at the end of treatment across both treatment groups. A study to evaluate the effect of high-dose RAS-antagonists (including zofenopril) and beta-blocker treatment for the primary prevention of cardiac events in a population of patients with T2D is recruiting (NCT02817360). The pharmacological properties of zofenopril indicate potential renoprotective ancillary features of the drug, which require confirmation through large-scale RCT specifically designed and conducted in patients at various stages of CKD. Losartan, an ARB, conferred salutary effects on renal and cardiovascular outcomes of patients with type 2 diabetes and nephropathy [28]. Losartan decreased the risk for doubling of Scr, ESRD, or death. Irbesartan exhibited similar renoprotective action for nephropathy attributed to type 2 diabetes as losartan [135]. Notably, this protection is independent of blood pressure control. Furthermore, the standard administration of ARBs was implemented, yet the progression of DKD continued to advance. The concurrent therapy of ACEI and ARBs was demonstrated to be even harmful for DKD [136]. The aforementioned novel therapies ultimately delivered disheartening consequences.

### 3.2. Sodium-Glucose Cotransporter 2 Inhibitors

The advent of SGLT2 inhibitors in 2014 sparked tremendous enthusiasm in strengthening the management of DKD. SGLT2 inhibitors are medications that have the potential to reduce the risk of ESRD, or death due to kidney disease, in individuals with T2D (Figure 3). This class of drug effectively blocks glucose absorption at the S1 segment of the proximal convoluted tubule, a process that accounts for the reabsorption of 90% of the glucose filtered by the glomerulus. This intervention results in glycosuria and, subsequently, improved glycemic control. SGLT2 inhibitors have been extrapolated to be involved in the progression of DKD via various mechanisms, including the activation of tubule-glomerular feedback, which may lead to a reduction in glomerular hyperfiltration. This process can mitigate the production of ROS and the formation of AGEs within proximal tubular cells. Additionally, it can reduce circulating inflammatory and fibrotic factors, such as TNF receptor-1, IL-6, MMP-7, and fibronectin-1, and decrease ketone production [137]. In light of RCTs, associated meta-analysis and systemic reviews, SGLT2 inhibitors are now commonly recommended for the treatment of most patients with DKD and an eGFR of ≥25 mL/min per 1.73 m^2^, irrespective of the glycemic management status [138,139]. The landmark trials such as the Canagliflozin and Renal Events in Diabetes with Established Nephropathy Clinical Evaluation (CREDENCE) and the Dapagliflozin and Prevention of Adverse outcomes in Chronic Kidney Disease (DAPA-CKD) trails have exerted a robust impact on the clinical application of SGLT2 inhibitors [140]. Notably, Canagliflozin had a 34% reduction in the death of renal events and a 32% decrease in the risk of ESRD in DKD patients [140]. Dapagliflozin has been shown to significantly reduce the risk of ESRD or death from renal events by 44%, as well as induce at least a 50% decline in eGFR. Additionally, it offers a 29% relative reduction in risk of death from CV events in CKD patients, irrespective of absence or presence of diabetes [141]. SGLT2 inhibitors are highly recommended in patients with severe albuminuria [142,143]. The Heart and Kidney Protection with Empagliflozin (EMPA-KIDNRY) trial was conducted to evaluate the impact of empagliflozin on CKD patients without diabetes. Empagliflozin revealed a lower rate of hospitalization from any cause by 14%, greater renal protective effects and a lower risk of death from cardiovascular events by 28%, with their efficacy being more conspicuous in those with ACR more than 300 mg/g [143]. Interestingly, the combined use of an SGLT2 inhibitor and ACEI/ARB shows a prominent decline in renal function approximately by 30–40%, surpassing the effects observed with ACEI/ARB alone [36]. Other potential mechanisms of action of SGLT2 inhibitors include enhancing cardiac function, and the decreased tubular transport associated reductions in oxygen consumption and oxidative stress. SGLT2 inhibitors act as a highly validated therapy for slowing the progression of DKD.

### 3.3. Nonsteroidal Mineralocorticoid Receptors Antagonists

In addition to the distal nephron, MRs are expressed on other cell types, including fibroblasts, macrophages, podocytes, and vascular cells. Decreased circulating plasma volume induces RAS activation [144], further promoting aldosterone secretion. This aldosterone then contributes to MR activation, resulting in sodium reabsorption and potassium excretion (Figure 3). The activation of MRs with high sodium intake leads to hypertension, contributing to glomerular damage and fibrosis [144]. Hyperglycemia, dyslipidemia, insulin resistance, and obesity upregulate the expression of MR, which elevates inflammatory (IL-1β, IL-6, TNF-α, MCP-1) and profibrotic factors (extracellular matrix proteins, PAI-1, TGF-β, CTGF), eventually resulting in the progression of DKD [145,146]. The upstream accumulation of renin due to long-term use of ACEI/ARB therapy can increase plasma aldosterone owing to “aldosterone escape”. Long periods of use of trandolapril showed an obvious increase in aldosterone in 40% of patients at 40 weeks with mounting albuminuria [147], which indicated that the combined use of ACEI/ARBs and MRAs might present optimal treatment for DKD.

As early as 2001, animal studies have revealed the therapeutic roles of MRAs in preventing the progression of DKD by reducing inflammation, fibrosis, and albuminuria [148]. Steroid-based MRAs, including spironolactone and eplerenone, have been commonly adopted for symptomatic heart failure patients (particularly with reduced ejection fractions) [149]. Additionally, they are highly effective in the treatment of primary hyperaldosteronism and refractory hypertension. Currently, NS-MRAs cannot serve as a substitute for steroidal MRAs in the treatment of heart failure and hyperaldosteronism. In spite of reduced albuminuria and blood pressure observed in DKD patients treated with MRAs, there are scarce clinical trials to verify these findings due to the high risk of hyperkalemia and reduction in kidney function. Unfortunately, there are no available data demonstrating that these MRAs reduce the risk of clinical outcomes. The utilization of steroidal MRA not only enhances the risk of hyperkalemia (by 2–3-fold) and acute kidney injury (by 2-fold), but also raises concerns about gynecomastia, a side effect associated with spironolactone. It is generally contraindicated to use MRAs in advanced kidney disease [150]. To further explore the benefits and risks of MRA treatment, additional trials are essential in diverse study populations. This includes examining patients with T2D but normal urine albumin excretion, individuals with T1D and CKD, those who have undergone a kidney transplant, patients with CKD but without T2D, and individuals undergoing dialysis.

The NS-MRAs, including finerenone, apararenone, esaxerenone, and ocedurenone, which distribute between heart and kidney tissue rather than influencing the kidney alone, are conspicuously different from steroidal-based MRAs [30]. Finerenone, a representative NS-MRA, has demonstrated a prominent reduction in albuminuria and blood pressure as well as risks of atherosclerotic disease and heart failure in the Finerenone in the Reducing Kidney Failure and Disease Progression in Diabetic Kidney Disease (FIDELIO-DKD) and Finerenone in Reducing cardiovascular mortality and morbidity in Diabetic Kidney Disease (FIGCARO-DKD) clinical trials in participants with T2D and DKD treated with ARB or ACEI as the standard care [151]. The FIDELIO-DKD trail has revealed that finerenone has demonstrated a more than 40% reduction in the risk of eGFR decline and a 18% reduction in death from renal disease, as well as a 14% reduction in prespecified secondary endpoints of death from CV events (heart failure, hospitalization, nonfatal stroke, or nonfatal myocardial infarction) [152], whereas it should be noted that finerenone still exert a slightly higher risk of hyperkalemia compared to the placebo (2.3% vs. 0.9%) [152]. The FIGCARO-DKD trail included patients with a higher risk of CV and less-advanced DKD. This trial further supported the benefits of finerenone in reducing CV causes. Finerenone has a high affinity and selectivity for MRs, as well as additional antagonist properties at the androgen receptor, which makes it more effective in blocking aldosterone binding to MRs, resulting in fewer side effects than older agents like spironolactone and eplerenone. Despite the risk of hyperkalemia, finerenone is well tolerated. Specifically, the FIDELIO-DKD and FIGCARO-DKD trials required serum potassium concentration of consistently 4.8 mmol/L during screening. While some participants had a slightly higher serum potassium of 4.9–5.0 mmol/L at randomization, selection was primarily based on a concentration of 4.8 mmol/L or lower. In the FIDELIO-DKD and FIGCARO-DKD trials, serum potassium levels were regularly monitored. One month after commencing the drug, the serum potassium levels were checked, as well as four months thereafter. Finerenone was continued with serum potassium ≤5.5 mmol/L. If serum potassium exceeded 5.5 mmol/L, the drug was temporarily withdrawn and serum potassium was reassessed in 72 h. Additionally, dietary potassium restriction and the use of concomitant medications, including diuretics and dietary potassium binders were allowed. Once potassium levels returned to ≤5.0 mmol/L, the drug was reintroduced.

The prespecified pooled analysis of FIDELIO-DKD and FIGCARO-DKD, referred to FIDELITY, included more than 13,000 participants with type 2 diabetes and across various stages of CKD and albuminuria. This analysis validated a 23% reduction in the risk of creatinine doubling, rapid renal function decline, RSRD, and death from renal disease. Additionally, there was a 14% decrease in the risk of the composite outcomes of CV [151]. However, it is important to note that FIDELITY revealed that the incidence of hyperkalemia was greater in spironolactone with resistant hypertension compared to those treated with finerenone (64.1% vs. 11.2%) [153]. A smaller phase 3 RCT clinical trial of Esaxerenone with Placebo in Japanese Type 2 Diabetic Patients with Microalbuminuria (ESAX-DN) failed to show a protective effect for the progression of DKD due to a short study duration with participants in early DKD or other unique characteristics of a single-country study [154]. An animal study showed that the combined use of finerenone and empagliflozin in hypertensive rats contributed to an obvious decrease in kidney fibrosis and albuminuria [155]. These findings suggest that this combination therapy may offer a promising approach for treating DKD. Nonetheless, a retrospective analysis of the DAPA-HF (Dapagliflozin in HFrEF) [156] and EMPEROR–Reduced trials (Empagliflozin Outcome Trial in Patients with Chronic Heart Failure with Reduced Ejection Fraction) [157] failed to validate the efficiency of the combination of these two drugs. It appears that the use of the SGLT2 inhibitor appears to counteract the hyperkalemia due to the addition of MRAs to ACEI or ARBs [158,159]. The 2022 guidelines issued by the American Diabetes Association (ADA) and the Kidney Disease Improving Global Outcomes (KDIGO) recommend the use of finerenone across a wide range of DKD patients with increased albuminuria despite treatment with an ACEI/ARB and SGLT2 inhibitor [124,160]. Finerenone is the only one approved for protection for cardiorenal events, whereas other NS-MRAs are primarily recommend for controlling blood pressure without conclusive outcome data in favor of their use in DKD. Esaxerenone has been observed to reduce albumin excretion. Nevertheless, the long-term renal and cardiovascular benefits of esaxerenone remain to be established, and its regulatory approval is not yet widespread.

### 3.4. Glucagon-like Peptide 1 Receptor Agonists

GLP, which is a peptide produced by the gut epithelium, has the potential to regulate blood glucose through activating the GLP-1R in the pancreas to lower glucagon and elevate insulin secretion. Incretin drugs, which include GLP-1RAs and dipeptidyl peptidase 4 (DPP4) inhibitors, have been developed to capitalize on these effects. GLP-1RAs have been approved for the management of hyperglycemia, prevention of atherosclerotic CV disease, and/or treatment of DKD patients at high risk for CV events, in spite of optimal therapy with metformin and SGLT2 inhibitors [161,162] (Figure 3). In addition, GLP-1RAs also help to promote weight loss, adding to their versatility in managing diabetes. A secondary analysis of glycemic lowering and CV outcome trails has confirmed the renoprotective actions of GLP-1RA in T2D through reducing albuminuria and slowing the decline in eGFR, independent of glycemic control [163,164]. GLP-1RAs have been reported to play reno-protective roles through ameliorating oxidative stress, cellular apoptosis, and fibrosis [161]. GLP-1RAs have the ability to curtail the generation of ROS and suppress the binding of NF-κB to its target gene, further reducing the downstream expression of cytokines (TNF-α, IL-1, IL-6) and fibrotic factors (TGF-β) [161]. Inhibition of NF-κB signaling by GLP-1RAs is a proposed mechanism that can effectively suppress the expression of proinflammatory cytokines and chemokines. A comprehensive review and network meta-analysis of RCTs demonstrated GLP-1RAs could reduce the risk of eGFR by 15 mL/min/1.73^2^ and the need to initiate renal replacement therapy by 22% within 5 years [165]. The Liraglutide Effect and Action in Diabetes: Evaluation of Cardiovascular Outcome Results (LEADER) revealed that liraglutide could lower the rate of new-onset persistent albuminuria [166]. Additionally, the primary evaluation of cardiovascular and other long-term outcomes with semaglutide in subjects with type 2 diabetes (SUSTAIN-6) showed that semaglutide could result in lower rates of new or worsening nephropathy [167]. A pooled analysis of SUSTAIN-6 and LEADER revealed that semaglutide and liraglutide contributed to a 24% reduction in albuminuria from the baseline to 2 years [33]. Semaglutide and liraglutide reduced the risk of sustained declines in eGFR to 40% and 50%, respectively [33]. Additionally, the recently published trail of the Effect of Efpeglenatide on Cardiovascular Outcomes (AMPLITUDE-O) revealed efpeglenatide resulted in a 32% reduction in the risk of composite renal outcomes independent of the baseline use of SGLT2 inhibitor [168]. In addition to lowering glucose, GLP-1RAs and DDP4 inhibitors can lead to a decrease in blood pressure and body weight [161]. Obesity has been associated with a decrease in adiponectin production and an increase in leptin levels. Leptin prompts the release of proinflammatory (IL-1, IL-6, TNF-α, MCP-1) and profibrotic (TGF-β, PAI-1, CTGF) factors [169]. Obesity also activates RAAS, which leads to higher intraglomerular pressure, eventually contributing to podocyte loss, progressive fibrosis, and renal failure [169,170]. DDP4 inhibitors have shown only modest improvement of albuminuria and have failed to delay the decline in eGFR [161]. Therefore, further research is needed to evaluate the long-term renal outcomes with these agents.

“The Effect of Semaglutide Versus Placebo on the Progression of Renal Impairment in Subjects with T2D and Chronic Kidney Disease” trail (FLOW) is the inaugural study to evaluate the impact of GLP-1RA on the primary kidney disease outcome [171]. Interim analysis has revealed unequivocal positive efficacy, leading to the early ending of the trial. It is anticipated that semaglutide will soon be recommended by KDIGO to slow the progression of DKD. Furthermore, the combined therapy of SGLT2 inhibitors and GLP-1RAs has been confirmed to reduce the risk of major adverse cardiac and cerebrovascular events, as well as heart failure in patients with type 2 diabetes [172]. KDIGO suggests the addition of GLP-1RA for patients who are already on metformin and SGLT2 inhibitor but have not achieved their glycemic control goals. The effect of a combination of these two drugs on the progression of DKD will be thoroughly evaluated using RCT in the near future.

### 3.5. Other Agents Exhibiting Potential Effectiveness on DKD

The nonspecific phosphodiesterase inhibitor PTF, which exhibits antiproliferative, anti-inflammatory, and antifibrotic roles, has been extensively studied in recent years [173]. In 2015, PTF was demonstrated to result in a smaller decrease in eGFR and a greater decline in residual albuminuria in patients with type 2 diabetes and stages 3–4 CKD under standard administration of RAS blockade [174]. A respective analysis of PTF was postulated to slow the progression of DKD through increasing the expression of soluble Klotho, which was associated with anti-inflammatory and antialbuminuric properties [175]. However, further rigorous trails of PTF need to be initiated to consolidate the reno-protective actions. ASK1 inhibitor has revealed protective effects on kidney injury through reducing inflammation and fibrosis in rodent models of DKD [78]. Additionally, a post hoc analysis of a phase 2 clinical trial of selonsertib, a selective ASK1 inhibitor, suggested selonsertib might be a potential therapeutic agent to prevent the progression of DKD despite the fact that the trail did not achieve the primary endpoint [79]. The multicenter Study Evaluating the Efficacy and Safety of Selonsertib in Subject with Moderate-to-Advanced Diabetic Kidney Disease (MOSAIC, NCT04026165) was completed before 2021. However, the data from this study has yet to be published. A phase 2 placebo-controlled trial of baricitinib, a JAK1/2 inhibitor, predominately reduced albuminuria and inflammatory factors (including intercellular adhesion molecule-1, plasma TNF receptor-1/2, and serum amyloid A) in patients with T2D and DKD [176]. Nonetheless, further trails need to be performed to investigate whether baricitinib can effectively prevent the progression of DKD. The JAK-STAT inhibitors have been used in the treatment of various immune-mediated diseases including psoriasis, spondyloarthritis, rheumatoid arthritis, and inflammatory bowel disease [177], supporting their potential therapeutic role in slowing the progression of DKD.

### 3.6. Lifestyle Affecting the Progression of DKD

In addition to screening for complications and management of cardiovascular risk factors in patients with DKD, lifestyle factors including smoking, diet, and physical activity play vital roles in the progression of DKD. Lifestyle modification should be an initial and essential intervention in the management of DKD. High dietary protein intake can lead to intraglomerular hypertension, which contributes to glomerular hyperfiltration, kidney damage and proteinuria [178]. Therefore, patients with advanced CKD are advised to restrict their potassium intake. Lower-potassium fruits, vegetables, and other foods are recommended for DKD patients, and the intake of vegetables and fruits should be in accordance with normal diabetic diet recommendations. Endogenous and dietary AGEs contribute to the progression of DKD. High dietary AGEs result in inflammation, insulin resistance, diabetes, and kidney injury [179]. Therefore, healthy diets, including fruits, vegetables, whole grains, legumes, fiber, unsaturated fats, plant-based proteins, and nuts have been revealed to be associated with lower incidences of CKD and albuminuria [180]. Additionally, restricting sodium intake is associated with a significant decrease in the risk of stroke, cardiovascular disease, and progression of CKD [181]. Obesity exerts harmful effects through various mechanisms, including insulin resistance, chronic inflammation and increased oxidative stress. Therefore, weight loss and maintaining a healthy body weight are important strategies for preventing and managing DKD. Lower levels of physical activity have been related to CVD [182]. Physical activity can reduce inflammatory markers, and improve endothelial function and insulin sensitivity [183]. Physical exercise contributes to a lower risk of CVD and CKD [184]. KDIGO recommends that patients with DKD engage in moderate-intensity exercise for a cumulative duration [13]. Tabacco is considered to be an explicit risk factor for the progression of DKD as well as secondhand smoke [185,186]. KDIGO recommends patients with DKD should quit smoking and avoid exposure to secondhand smoke [13].

## 4. The Value of Multidisciplinary Treatment and Drug Combination Therapy in Clinical Application

For a long period of time, RAS inhibitors were the only available drugs to treat DKD, and no other specific medications existed. The Japanese Diabetes Optimal Integrated Treatment Study for three major risk factors of cardiovascular disease (J-DOIT3), encompassing 2542 patients with T2D, assigned a targeted HbA1c level of 6.2%, which failed to significantly reduce cardiovascular events but had a positive impact on delaying the progression of DKD [187]. Multidisciplinary treatment, including blood glucose control, blood pressure control with RAS inhibitor, lipid control, and lifestyle modifications, significantly suppressed renal events.

Each drug class, when coupled with an RAS blockade, has shown protective effects on kidney and cardiovascular events. When managing heart failure, a combination of three or four drugs may be important for reducing cardiorenal events. Practice guidelines articulate that an RAS blockade should be maximally tolerated before adding other medications (SGLT2 inhibitors, NS-MRAs, and GLP-1RAs) [160]. SGLT2 inhibitors and finerenone have been revealed to lead to lesser mitigating of renal function decline [152,188,189]. An animal study further confirmed that combined treatment of empagliflozin and finerenone [155] resulted in a decrease in blood pressure, proteinuria, plasma creatinine, uric acid, vasculopathy, cardiac fibrosis, and mortality even when the eGFR decreased to 25 mL/min/1.73 m^2^ [155]. The combination of dapagliflozin and steroidal MRA eplerenone showed additional reduction in albuminuria and risk of hyperkalemia compared with the use of eplerenone alone [190], aligning with the lower incidence of hyperkalemia when an SGLT2 inhibitor is combined with finerenone from the FIDELIO-DKD trial [158].

The FIDELITY subgroup analysis further revealed that finerenone showed greater cardiorenal benefits regardless of whether an SGLT2 inhibitor or GLP-1RA was used in combination at baseline or any time during the trail [158]. However, no studies have evaluated the simultaneous use of all four agents in heart failure or compare the combined use of different drugs against each other. Each drug class that consolidated improved outcomes was combined with an RAS blockade in DKD patients. The study to investigate the combination effect of finerenone and empagliflozin in participants with chronic kidney disease and type 2 diabetes using a UACR endpoint study (CONFIDENCE) is ongoing to evaluate whether combined treatment of finerenone and the SGLT2 inhibitor outshines each drug alone [191]. A powered study evaluating the efficiency and safety of the combined therapy of four different agents is still needed.

## 5. Biomarkers and Future Therapeutic Targets for DKD

### 5.1. Molecule and Molecular Pathways as Biomarkers

The molecular mechanisms involved in the onset and progression of DKD are intricate. It is crucial to consider how each molecule and pathway influence DKD, particularly in humans. Albuminuria, together with eGFR, remains valuable for monitoring kidney function. Novel biomarkers in the field of DKD are urgently required to enhance early prevention and prognostic accuracy. Epidemiologic studies have identified inflammation and fibrosis markers as independent predictors of CKD progression in T1D and T2D. Serum galectin-3, which plays a role in promoting fibrosis in both the kidney and heart, has been independently linked to serum creatinine doubling and the development of albuminuria in individuals with T2D [192]. In a case-control study in patients with type 2 diabetes, elevated plasma levels of chemokine ligand-16 (CXCL-16), angiopoietin-2, and TGF-β1, which are systemic biomarkers of inflammation, fibrosis and endothelial dysfunction, are independently associated with the development of microalbuminuria [193]. TNF-a, which is produced by activated macrophages and resident kidney cells within the glomerulus and tubules, triggers the release of other cytokines, chemokines, apoptosis, and cytotoxic effects [194]. Furthermore, the clinical activation of TNF receptor-1 (TNFR1) exhibits a significant correlation with both kidney and cardiovascular outcomes in individuals with diabetes [195]. In addition, the inflammatory factors, including IL-1, IL-16, IL-18, MCP-1, MMP-9, PAI-1, and CTGF, have also been implicated in the pathogenesis of DKD. These cytokines are thought to play a role in the development and progression of DKD by promoting inflammation and fibrosis in the kidneys. In individuals with diabetes, circulating C-X-C motif ligands (CXCL9 and CXCL10) levels are significantly elevated, which have been associated with the recruitment of T helper cells 1 and 17 into the kidney [196]. These serum markers have the potential to enhance renal risk assessment models beyond traditional clinical factors.

In contrast to kidney biopsies, urine is effortlessly attainable, making the detection of biomarkers within this bodily fluid an invaluable asset in the diagnosis and management of kidney disease. In patients with T2D, TNF-α levels in urine, but not in serum, are linked to the occurrence and severity of microalbuminuria [194]. The urinary levels of CXCL10 increase significantly in individuals with diabetes [196]. The urinary levels of CXCL10 are found to be reduced in phase 2 RCT of baricitinib in individuals with T2D and DKD [176]. These studies may indicate that CXCL10 is associated with development of DKD. CKD273, a urinary biomarker pattern that indicates fibrosis due to the presence of collagen fragments, can also predict the progression of CKD in individuals with diabetes. The high-risk CKD273 score was strongly associated with an increased risk of developing albuminuria in individuals with T2D, even after adjusting for traditional CKD risk factors [197].

Certain circulating lncRNAs have been demonstrated to possess remarkable potential as sensitive and reliable biomarkers for the early diagnosis or prognosis of DKD, or as therapeutic targets for slowing the progression or even inducing regression of established DKD. *MALAT1* is commonly present in glomerular podocytes, renal tubular cells, and macrophages, which plays a distinct role in various pathogenic processes that lead to the development of DKD [198]. This unique characteristic can also be applied to other lncRNAs, such as *PTV1*, *ERBB4*-IR, and nuclear enriched abundant transcript-1 (*NEAT1*) [117]. *PTV1* is the first noncoding RNA that has been reported to be linked to kidney disease. *MALAT1*, *PTV1*, *ERBB4*-IR, and *NEAT1* all play significant roles in promoting the accumulation of ECM in DKD by targeting the TGF-β1 pathway.

### 5.2. Future Treatment for DKD

Hypoxia-inducible factor prolyl hydroxylase inhibitor. The exposure of tubular cells to hypoxia due to reduced blood flow triggers tubular cell apoptosis and the secretion of cytokines, particularly TGF-β, which in turn activates interstitial fibroblasts and enhance the production of the extracellular matrix, ultimately leading to the progression of tubulointerstitial fibrosis. Tubulointerstitial fibrosis is also known to diminish the efficiency of oxygen diffusion, possibly due to the dense accumulation of fibrotic tissue within the kidney’s tubulointerstitial space [199]. This can lead to a decrease in oxygen availability to the surrounding tissues, further exacerbating kidney function decline. The hypoxia-inducible factor (HIF) genes include the genes coding erythropoietin (EPO), glycolytic enzymes, and vascular endothelial growth factor-A. HIF is a pivotal transcription factor that mediates the organism’s adaptation to oxygen deprivation. The HIF-prolyl hydroxylase (HIF-PH) inhibitor effectively blocks HIF-α degradation by inhibiting HIF-PH, which is responsible for HIF-α oxygen-dependent degradation. This not only stabilizes HIF-α expression, but also further activates it. The long-term observation of ischemia-reperfusion injury (AKI-to-CKD transition model) was reported to mitigate renal fibrosis and prevent anemia through the administration of the HIF-PH inhibitor [200]. Activating HIF can mitigate tubular interstitial injury through suppressing tubular cell apoptosis and inflammatory responses in Thy-1 nephritis and 5/6 nephrectomy [201,202]. Whether HIF is beneficial for tubular cell regeneration remains controversial. It is well established that HIF promotes the expression of stromal-derived factor-1 and progenitor cells to damaged tissues [203]. With regard to cell regeneration and proliferation, HIF activation has been associated with the induction of p27, which can potentially repress the cell cycle [204]. A significant concern regarding the long-term use of these agents is their potential impact on tumor growth. A study on long-term effects of HIF activators in a 5/6 nephrectomy model found that renal fibrosis was exacerbated in the long-term (2 to 12 weeks) group [205]. It is worth noting that the application of a HIF-PH inhibitor significantly reduced albuminuria and glomerular inflammation in a mouse model of T2D [206].

In conclusion, the long-term impact of HIF-PH inhibition or HIF activation on CKD is likely to be influenced by various factors, including the primary disease and its stage. Therefore, further exploration is required through ongoing clinical trials and additional fundamental research. The HIF-PH inhibitors have been approved for renal anemia in CKD patients not yet on dialysis. However, further clinical trials are essential to evaluate their efficacy as a treatment for CKD, including DKD, in the future.

AGE inhibitor. AGE is a product of nonenzymatic protein and nucleic acid glycation [207]. Hyperglycemia and oxidative stress could induce the accumulation of AGE, leading to damage in various organs. AGE accumulation has been observed to be associated with the progression of DKD in human kidney samples [208,209]. AGE accumulation has been associated with the development of DKD symptoms in the kidneys of healthy rats, including mesangial expansion, glomerular basement membrane thickening, glomerular hypertrophy, glomerular sclerosis, and albuminuria [94]. RAGE overexpression in diabetic mice was reported to exacerbate DKD histological changes and accelerate renal dysfunction progression [210]. AGE, whether directly or through its receptor RAGE, can significantly enhance oxidative stress and inflammation through NF-κB activation, which promotes fibrosis by inducing TGF-β expression. These effects have been observed in various experimental models and suggest a potential role for AGE in the pathogenesis of CKD [211,212].

A number of clinical trials have been conducted on AGE inhibitors, yet their efficacy remains controversial. Pyridoxamine’s impact on serum creatinine levels and urinary TGF-β1 excretion was found to be significantly reduced after six months of treatment in patients with DKD [213]. The administration of thiamine was revealed to effectively reduce albuminuria in patients with DKD [214]. In the trial, Aminoguanidine Clinical Trial in Overt Nephropathy (ACTION), aminoguanidine also reduced proteinuria and inhibited GFR decline [215]. In contrast, the Aminoguanidine Clinical Trial in Overt Type 2 Diabetic Nephropathy (ACTION II) was terminated in patients with DKD due to the ineffectiveness of aminoguanidine, which was accompanied by adverse effects such as anemia, vitamin B6 deficiency, and liver dysfunction. When benfotiamine was administered to DKD patients, there was no significant difference in albuminuria compared to the placebo group [216]. On the contrary, it has been reported that RAGE inhibitors can prevent the progression of DKD in rats [88].

AGE accumulation is a key component of metabolic memory, making its inhibition an alluring therapeutic approach. Although some trials have not demonstrated the efficacy of AGE inhibitors in treating DKD, there is still scope for improvement in terms of optimal administration and sample sizes. A large RCT with optimal administration of AGE inhibitors is anticipated to establish their efficacy in the treatment of DKD.

Epigenetic regulator. Epigenetics is a DNA sequence-independent regulatory mechanism of gene expression, involving histone modification, DNA methylation, and noncoding RNA. Epigenetic alterations, even those resulting from hypoxia or temporary hyperglycemia, are recognized as being stored within cellular memory, ultimately causing irreversible renal damage [115]. In fact, it has been reported that vascular endothelial cells exposed to hyperglycemia continue to increase oxidative stress and trigger inflammation, even after blood glucose levels have normalized [217]. The altered histone modifications in tubular cells and aberrant DNA methylation in mesangial cells have been revealed to involve the progression of diabetic nephropathy [218,219,220]. Therefore, epigenetic genetic modifications play a pivotal role in the progression of DKD.

Histone modification inhibitors are being investigated as a potential therapy for addressing these epigenetic alterations. The histone deacetylase inhibitors, including valproate, sodium butyrate, vorinostat, and trichostatin, have been revealed to effectively reduce proteinuria and mitigate oxidative stress, inflammation, fibrosis, and glomerular damage in the DKD rat model [221]. Furthermore, dznep, a specific inhibitor of Ezh2, H3K27 methyltransferase, was revealed to effectively prevent renal fibrosis in mice models of UUO and the transition from AKI to CKD [222]. In addition, treatment with MM-102, the inhibitor of mixed-lineage leukemia 1(MLL1) or myeloid/lymphoid, H3K4 methyltransferase, has been found to effectively prevent fibrosis in mice undergoing a transition from AKI to CKD after ischemia-reperfusion injury [223].

In terms of DNA methylation, research has shown that the promoter region of thioredoxin-interacting protein (TXNIP) is hypomethylated in whole blood cells and blood monocytes of patients with DKD [224]. The deletion of TXNIP was revealed to mitigate renal damage in the DKD mouse model [225]. This hypomethylation may potentially contribute to the progression of advanced DKD. In terms of histone modification, the report reveals that there is an increase in H3K9 acetylation (H3K9ac) within the promoter region of the high-glycated-hemoglobin (Hb A1c) group, regarding the histone modification status of blood lymphocytes and monocytes derived from individuals with T1D [226]. H3K9ac has been established as being linked to the NF-κB pathway, indicating that heightened levels of H3K9ac may play a role in the progression of DKD through the inflammatory pathway [227]. Treatment of mesangial cells with losartan under hyperglycemic conditions was revealed to mitigate the increase in H3K9/14ac in the promoter regions of PAI-1, RAGE, and MCP-1 [228], suggesting a close correlation between AGEs and epigenetics in the development of metabolic memory. Lipids are also known to trigger the activation of SET7, an H3K4 methyltransferase, ultimately promoting gene expression that leads to glomerular hypertrophy and renal fibrosis [229]. HIF-1, the master regulator of the hypoxic response, recruits histone-modifying enzymes, particularly histone demethylases such as lysine demethylase (KDM) 3A, KDM3B, and KDM3C, to its binding sites [230]. These enzymes modify the chromatin structure and regulate gene expression. Human umbilical venous endothelial cells experienced a remarkable physiological shift upon exposure to hypoxic stimuli. HIF-1, the protein responsible for adapting cells to oxygen-poor environments, bounds specifically to the upstream regulatory region of the gene encoding *GLUT3* [231]. This interaction not only altered the transcriptional initiation of GLUT3 but also recruited KDM3A, a histone demethylase, to the site. This recruitment is crucial, as it allows for the fine-tuning of gene expression in response to environmental stimuli.

NcRNA is a generic term for RNAs that are not translated into proteins. Aspartyl-tRNA synthetase antisense 1 (DARS-AS1), a lncRNA, was activated by HIF-1 in tubular cells under hypoxic conditions and exerted an anti-apoptotic effect on tubular cells [232]. NcRNAs are particularly associated with DKD, especially miRNA-21 and miRNA-29. MiRNA-21 is particularly abundant in the kidney and has a close relationship with TGF-β signaling [233]. Seminal studies have emphasized the remarkable potential of noncoding RNAs as innovative therapeutic targets. In 2015, a phase II random control trails (RCT) of teprasiran, which was a siRNA (QPI-1002) and was designed to restrict p53-induced apoptosis, revealed that its administration could reduce the incidence, severity, and duration of early acute kidney injury (AKI) in high-risk patients undergoing cardiac surgery at day 90 [234]. However, a phase III study of teprasiran was prematurely terminated at one-year follow-up due to its inability to meet the efficacy outcomes at day 90 (NCT03510897). Almost at the same time, a phase III trial was conducted to evaluate the use of QPI-1002 in preventing a delayed graft function in kidney transplant recipients from older donors (NCT02610296). The trial was successfully completed in 2020, yet the data remains undisclosed. Anti-miR-192 was revealed to improve the renal structure and fibrosis in DN mice [235,236]. Though there are no clinical trials for their use in treatment for DKD, the use of noncoding RNA brings new hope.

CKD, particularly DKD, is widely acknowledged to be irreversible once it has advanced beyond a certain stage. If the epigenetic changes in CKD can be fully understood, there is potential to develop innovative drugs that can reverse the progression of the disease. Current research on epigenetic modification strongly suggests this possibility.

## 6. Limitations of the Study

Although we are trying to delve into the intricate mechanism underlying DKD, as well as current treatment options, biomarkers and future research direction for DKD, it is acknowledged that this study is rooted in the previous research, which may be unable to reflect the latest achievements in the field. DKD is challenging to diagnose in its early stage due to the absence of specific biomarkers, such as albuminuria and eGFR, additional research is needed to identify more specific and sensitive biomarkers for the early diagnosis of DKD. Furthermore, current treatment options available for DKD are limited, and their therapeutic effects are often unsatisfactory. We have also provided potential therapeutic directions for DKD, but further clinical trials are required to validate their efficacy.

## 7. Conclusions

DKD is featured in a range of hemodynamic, inflammatory and metabolic process, ultimately converging on the fibrotic pathway. Even if glycemia is normalized, DKD may be propagated due to persisted expression of proinflammatory and profibrotic mediators. Lifestyle and epigenetics are also associated with the progression of DKD. Despite the emerging of RAS blockades, SGLT2 inhibitors and NS-MRAs, current treatment options are limited in their ability to effectively impede kidney disease progression and abate risks of comorbidities and death among patients with DKD. RAS blockers, SGLT2 inhibitors and NS-MRAs have shown great efficacy in reducing the risk of renal disease [237]. However, patients vary in their response to RAS blockades. The pharmacodynamic responses to SGLT2 inhibitors decrease with elevating severity of renal damage, and the incidence of hyperkalemia increases in the treatment of NS-MRAs. Other agents targeting Nrf2, fibrosis, PKC, EA, ERA, renin, glycosaminoglycan polysaccharide, and phosphodiesterase achieved unmet consequences. Plenty of residual risks of progression for DKD persist. Thus, effective therapy for DKD is yet unrealized. Just recently, Novo Nordisk announced that they would stop their semaglutide kidney outcome trail due to clear protective roles on the basis of interim analysis. With the advent of GLP-1RAs, the possible fourth class brings new efficacious treatment for effectively arresting the progression of DKD. The combined therapy of these drugs holds promise for improving outcomes in patients with DKD. However, starting combined medication requires significant consideration. The difficulty in managing DKD lies in the challenges associated with early diagnosis, mainly due to the absence of specific and sensitive biological indicators. We have summarized several biomarkers from the existing literature, including inflammatory and fibrotic markers, and lncRNAs. Inflammatory markers can indicate the inflammatory response when its levels are elevated in DKD. Fibrosis markers, such as TGF- β, could reflect the extent of fibrosis, which is important for early diagnosis and prognostic evaluation of DKD. LncRNAs are a new class of biomarkers for early diagnosis and prognosis due their stable ability.

We present a comprehensive overview of the latest advancements in basic and clinical research that have illuminated the molecular mechanisms underlying DKD, along with potential therapeutic interventions. Future intervention including HIF inhibitors, anti-inflammatory agents, AGE inhibitors, and epigenetic modification may provide novel treatment options for DKD. To further enhance our understanding of the pathogenesis of DKD and establish effective treatment strategies, it is imperative to conduct extensive research.

## Data Availability

Not applicable.
